# Barriers to access and utilization of healthcare by children with neurological impairments and disability in low-and middle-income countries: a systematic review

**DOI:** 10.12688/wellcomeopenres.16593.1

**Published:** 2021-03-18

**Authors:** Lucy W. Mwangi, Jonathan A. Abuga, Emma Cottrell, Symon M. Kariuki, Samson M. Kinyanjui, Charles RJC. Newton

**Affiliations:** 1 Clinical Research (Neurosciences), Kemri-Wellcome Trust Research Programme, Kilifi, PO Box 230-80108, Kenya; 2 Global Child Heath Group, Emma Children’s Hospital, Academic Medical Center, University of Amsterdam, Amsterdam, AHTC, Tower C4, Paasheuvelweg 25 1105 BP Amsterdam, The Netherlands; 3 Department of Psychiatry, University of Oxford, Oxford, Oxford OX3 7JX, UK; 4 Nuffield Department of Medicine, University of Oxford, Oxford, Oxford OX3 7BN, UK

**Keywords:** healthcare, neurological impairments, disability, rehabilitation, resource-limited settings

## Abstract

**Background: **Neurological impairments (NI) and disability are common among survivors of childhood mortality in low-and middle-income countries (LMICs). We conducted a systematic review to examine the barriers limiting access and utilization of biomedical care by children and adolescents with NI in LMICs.

**Methods: **We searched PubMed, Latin America and Caribbean Health Sciences Literature, Global Index Medicus, and Google Scholar for studies published between 01/01/1990 and 14/11/2019 to identify relevant studies. We included all reports on barriers limiting access and utilization of preventive, curative, and rehabilitative care for children aged 0-19 years with NI in five domains: epilepsy, and cognitive, auditory, visual, and motor function impairment. Data from primary studies were synthesized using both qualitative and quantitative approaches, and we report a synthesized analysis of the barriers identified in the primary studies.

**Results: **Our literature searches identified 3,074 reports of which 16 were included in the final analysis. Fourteen studies (87.5%) originated from rural settings in sub-Saharan Africa (SSA). Factors limiting access and utilization of healthcare services in >50% of the studies were: financial constraints (N=15, 93.8%), geographical inaccessibility (N=11, 68.8%), inadequate healthcare resources (N=11, 68.8%), inadequate education/awareness (N=9, 56.3%), and prohibitive culture/beliefs (N=9, 56.3%). Factors reported in <50% of the studies related to the attitude of the patient, health worker, or society (N=7, 43.8%), poor doctor-patient communication (N=5, 31.3%), physical inaccessibility (N=3, 18.8%), and a lack of confidentiality for personal information (N=2, 12.5%). Very few reports were identified from outside Africa preventing a statistical analysis by continent and economic level.

**Conclusions: **Financial constraints, geographic inaccessibility, and inadequate healthcare resources were the most common barriers limiting access and utilization of healthcare services by children with NI in LMICs.

**PROSPERO registration:** CRD42020165296 (28/04/2020)

## Introduction

Globally, at least 300 million children live with some form of neurological impairment (NI) or disability, of which >90% originate from low- and middle-income countries (LMICs) ( Olusanya *et al.*, 2020). These children, especially those with moderate, severe or multiple disabilities, are vulnerable to infections and accidents which might necessitate hospitalization and subsequent rehabilitation ( Moreau *et al.*, 2013). However, numerous factors limit access or utilization of available preventive, curative, and rehabilitative services by these children ( WHO, 2011).

Children with NI require regular access to preventive, curative, and rehabilitative services. Primary preventive services such as nutritional supplementation and immunization have an established role in the prevention of NI ( Groce *et al.*, 2014). Vitamin A supplementation and early childhood immunization against measles, rubella, and poliomyelitis substantially reduce the risk of developing NI ( Maulik & Darmastadt, 2007), but also reduce morbidity in those that have NI. Secondary prevention involves early screening to identify those already with NI for treatment (or management) to alter the prognosis. Diagnostic tests such as the electroencephalogram (EEG) may help classify seizures and determine treatment ( Bassili *et al.*, 2002). Tertiary and quaternary prevention includes treatment and rehabilitation, respectively, to prevent premature mortality, improve functioning, and quality of life. Corrective surgery for children with hearing impairments ( Roland *et al.*, 2016) and antiepileptic drugs for children with epilepsy ( Mbuba *et al.*, 2012) are widely documented curative/management options. Rehabilitative services to reduce activity limitation and to improve participation in respective communities include physiotherapy and occupational therapy. However, children from LMICs lack adequate access to the aforementioned continuum of healthcare due to multifarious barriers such as geographical inaccessibility, societal stigma, and financial constraints ( WHO, 2011).

**Figure 1.**
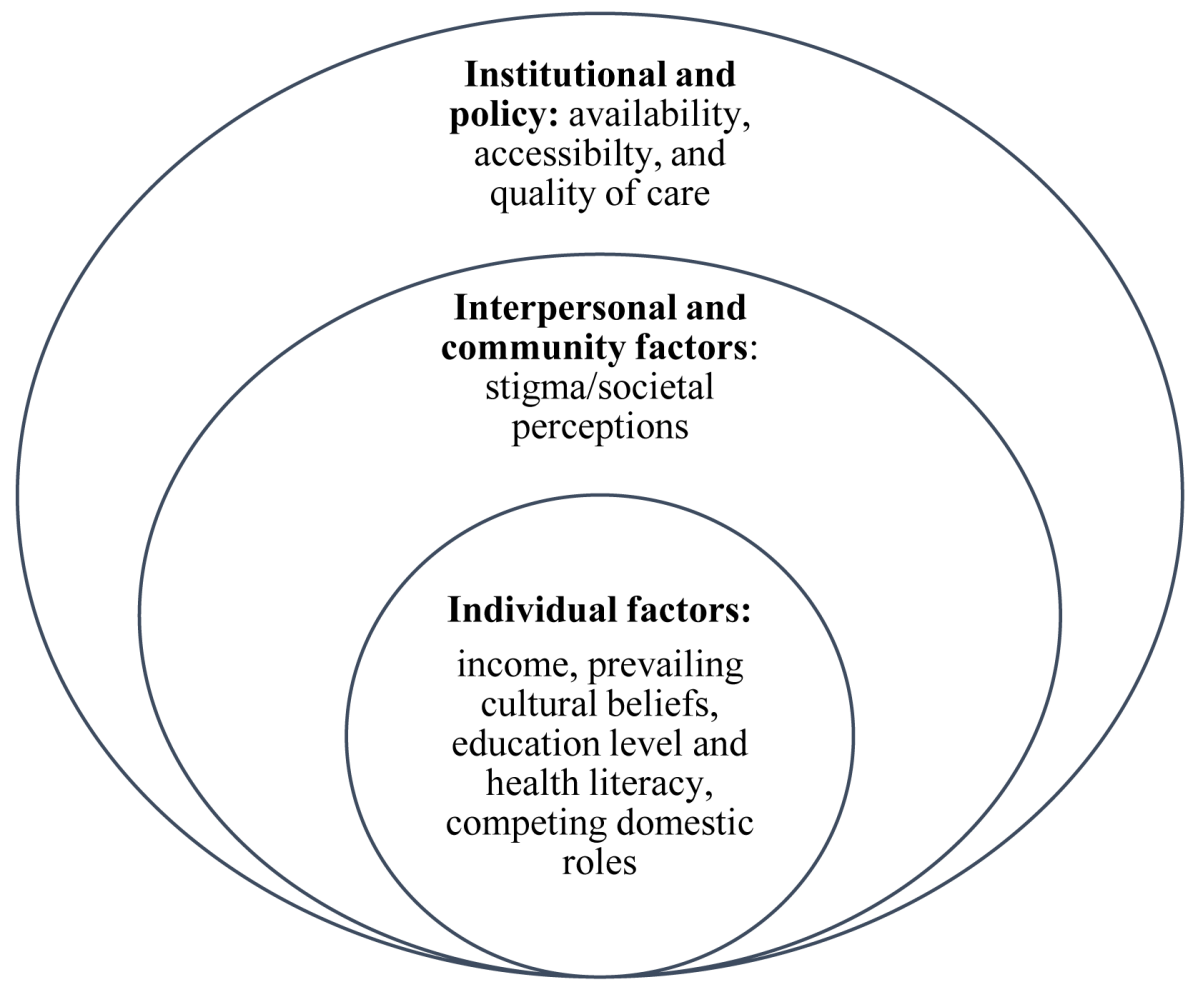
Modified socio-ecological model with layers of barriers limiting access/utilization of healthcare by children with neurodisability.

A range of barriers, both from the consumer’s and provider’s perspectives, may hinder uptake of biomedical services by affected children in LMICs ( Figure 1). Poverty in families of children with NI and inadequate government funding limits the prioritization, availability, access, and quality of rehabilitative care ( Bright *et al.*, 2018). Besides, prevailing cultural beliefs and societal perceptions shape caregivers’ perceptions especially on the aetiology of NI, which affects decisions about the alternatives of care, and ultimately the prognosis of neurodisability ( Zuurmond *et al.*, 2019). A 2015 report published by the World Health Organization (WHO) further highlights that people with NI usually experience discrimination at the point of care, which may discourage subsequent seeking of appropriate services ( WHO, 2015). Measures such as decentralizing health systems have been proposed to bring services closer to the people and to reduce the geographical distance (distance decay) especially for rural-dwelling populations ( Saltman *et al.*, 2007).

Some studies from LMICs have separately identified contextual factors that limit access and utilization of existing biomedical services by children with NI. The available evidence on barriers preventing the use of preventive, curative, and rehabilitative services by children with NI in LMICs is fragmented. We, therefore, conducted a systematic review to identify and classify barriers limiting access and utilization of biomedical services by children and adolescents with NI in LMICs settings. Synthesized evidence from LMICs is required to inform policy and public health action to ensure equity in access and utilization of healthcare as enshrined in the agenda of the United Nation’s sustainable development goals ( Pettigrew *et al.*, 2015; Tangcharoensathien *et al.*, 2015).

## Methods

### Reporting guidelines

We used the National Health Service Centre for Review and Dissemination (CRD) recommendations ( Booth *et al.*, 2010) and the preferred reporting items for systematic reviews and meta-analyses (PRISMA) guidelines ( Abuga *et al.*, 2021; Moher *et al.*, 2009) to conduct this systematic review. We registered a protocol with the International Prospective Register for Systematic Reviews (PROSPERO), registration ID CRD42020165296 (28 ^th^ April 2020).

### Search strategy and inclusion criteria

**Figure 2.**
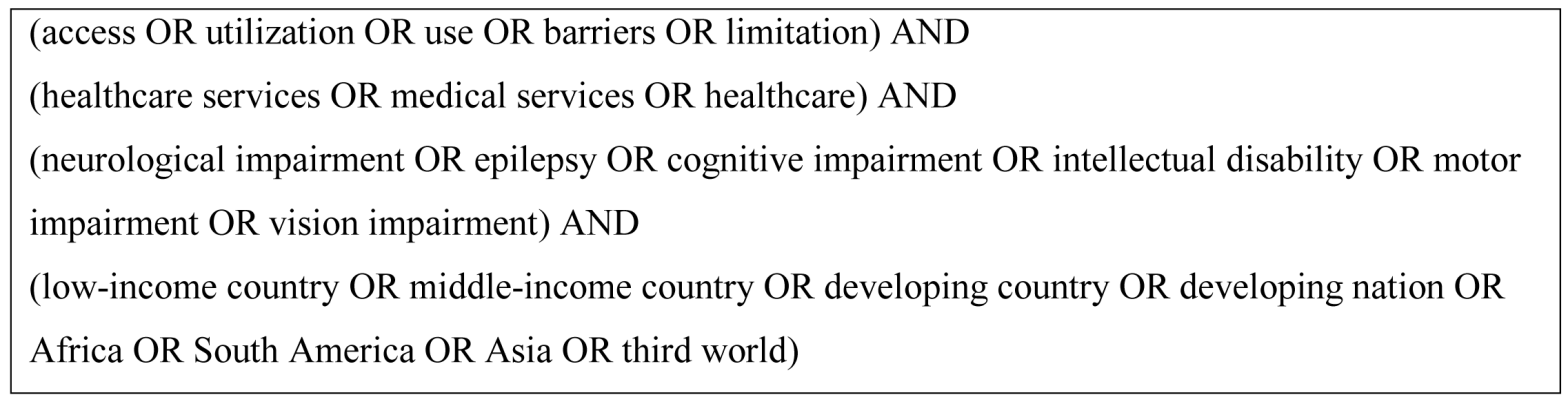
Search terms used in the systematic review.

We searched PubMed, Latin America and Caribbean Health Sciences Literature ( LILACs), Global Index Medicus, and Google Scholar databases for reports published between 01/01/1990 and 14/11/2019 to identify relevant reports. These searches covered a period through which the burden of NI and disability has significantly increased, globally ( Global Research on Developmental Disabilities Collaborators, 2018). The key search terms were ‘neurological impairment’ and ‘access’ or ‘utilization’ and ‘healthcare services’ as shown in Figure 2, with limits to human studies only.

Eligible studies included those of: (i) children and adolescents aged 0–19 years; (ii) children or adolescents with NI in five domains namely, epilepsy, and impairments in cognitive, hearing, visual, and motor functions; (iii) assessing access and utilization of healthcare services including preventive, curative, and rehabilitative care; and (iv) LMICs as defined by the World Bank ( World-Bank, 2019). We excluded systematic reviews, reports, studies on adults, studies conducted in HICs, commentaries, and studies published in languages other than English.

### Defining neurological impairment and disability

Neurological impairment was defined as a deficit of the central nervous system (CNS) resulting in functional limitation in five domains namely: epilepsy, and cognitive, hearing, visual, and motor impairments ( Mung'ala-Odera *et al.*, 2006). Epilepsy was defined according to the International League Against Epilepsy’s (ILAE) guidelines as the presence of two or more unprovoked seizures occurring more than 24 hours apart within the previous 12 months ( Fisher *et al.*, 2014; Thurman *et al.*, 2011). A child with moderate or severe cognitive impairment refers to a child with a z-score below-2SD or -3SD, respectively based on neuropsychological scores standardized to the normal population. Moderate motor impairment is defined as a difficulty in holding objects, dressing and sitting upright, or ambulant only with help, while severely impaired children include those unable to walk or have no functional use of the hands ( WHO, 2011). Moderate hearing impairment refers to a 41–70dB loss in the best ear or difficulty in hearing with a hearing aid; severe impairment refers to greater than 70dB hearing loss or complete loss of hearing in the best ear ( WHO, 2020). A child with moderate vision impairment has a visual acuity poorer than 6/18 while those with visual acuity poorer than 6/60 meters are classified as having severe vision impairment ( WHO, 2019). There was a variation in the actual definitions used in the individual studies and most reports lacked information on NI severity. We, therefore, used these definitions as a formal guideline to verify the actual definitions used in the included reports.

### Study selection, data extraction, and quality appraisal

Study selection was done in two phases. In the first phase, two reviewers (LM and JA) independently screened the reports identified by the searches by title and abstract for eligibility. In the second phase, both reviewers (LM and JA) examined the full-text of articles obtained from the first phase against the inclusion and exclusion criteria. Disagreements were resolved through consensus in discussions involving three reviewers (LM, JA and EC). We extracted data relevant for analysis using a pretested data extraction tool designed by the reviewers using guidelines from the PRISMA checklist. Extracted data included author details, study setting, study population, participant characteristics, the type of healthcare services sought, and barriers hindering access or utilization of the services by children with NI or disability. We assessed the quality of each study using the Joanna Briggs Institute (JBI) critical appraisal tools, which are distinct for case-control, cohort, and qualitative studies ( Munn *et al.*, 2014).

### Synthesis of included reports

Eligible reports identified by the searches and the selection processes were both qualitative and quantitative in design. We grouped all eligible studies based on the domain of NI investigated, and then listed the barriers limiting access and use of biomedical services as identified by these studies. Services sought by children with NI were classified as preventive, curative, or rehabilitative. The barriers identified from all eligible studies were classified, analysed, and reported based on seven all-inclusive themes namely: financial constraints, geographical inaccessibility, the inadequacy of healthcare resources, inadequate education/awareness, prohibitive culture/beliefs, competing domestic roles, and a lack of confidentiality/anonymity ( Table 1). 

**Table 1.** Classification of barriers preventing access/utilization of healthcare by children with neurological impairments/disability.

Theme (category) of barrier	Criteria for inclusion
1. Financial constraints	Determined by whether the patient/caregiver was able to pay for biomedical services and all indirect costs incurred while seeking care.
2. Education/ awareness	Lack of/inadequate information or awareness from healthcare facilities about the availability of care services; or caregiver/patient unable to seek services due to limited education/information/health illiteracy.
3. Culture/beliefs	Caregivers or patients’ values and perceptions inhibiting seeking biomedical services or societal attitude influencing health-seeking behaviour
4. Geographical inaccessibility and physical barriers	Geographical inaccessibility described as the proximity of the healthcare facilities from the patient’s/caregiver residence. Inaccessibility also included the inability to access healthcare services due to physical disabilities and/or unfavourable infrastructural design of healthcare facilities or transport systems.
5. Inadequate healthcare resources and quality of care	The attitude of healthcare workers and availability of appropriate services/equipment such as expert consultation, clinical assessment, supply of critical drugs, laboratory equipment, and testing in the healthcare facilities.
6. Confidentiality/anonymity	Comprising privacy and protection of patient information of those seeking healthcare.
7. Childcare/competing domestic roles	Domestic roles such as childcare or taking care of the sick and elderly at home as competing roles for the caregivers.

## Results

### Search results

**Figure 3.**
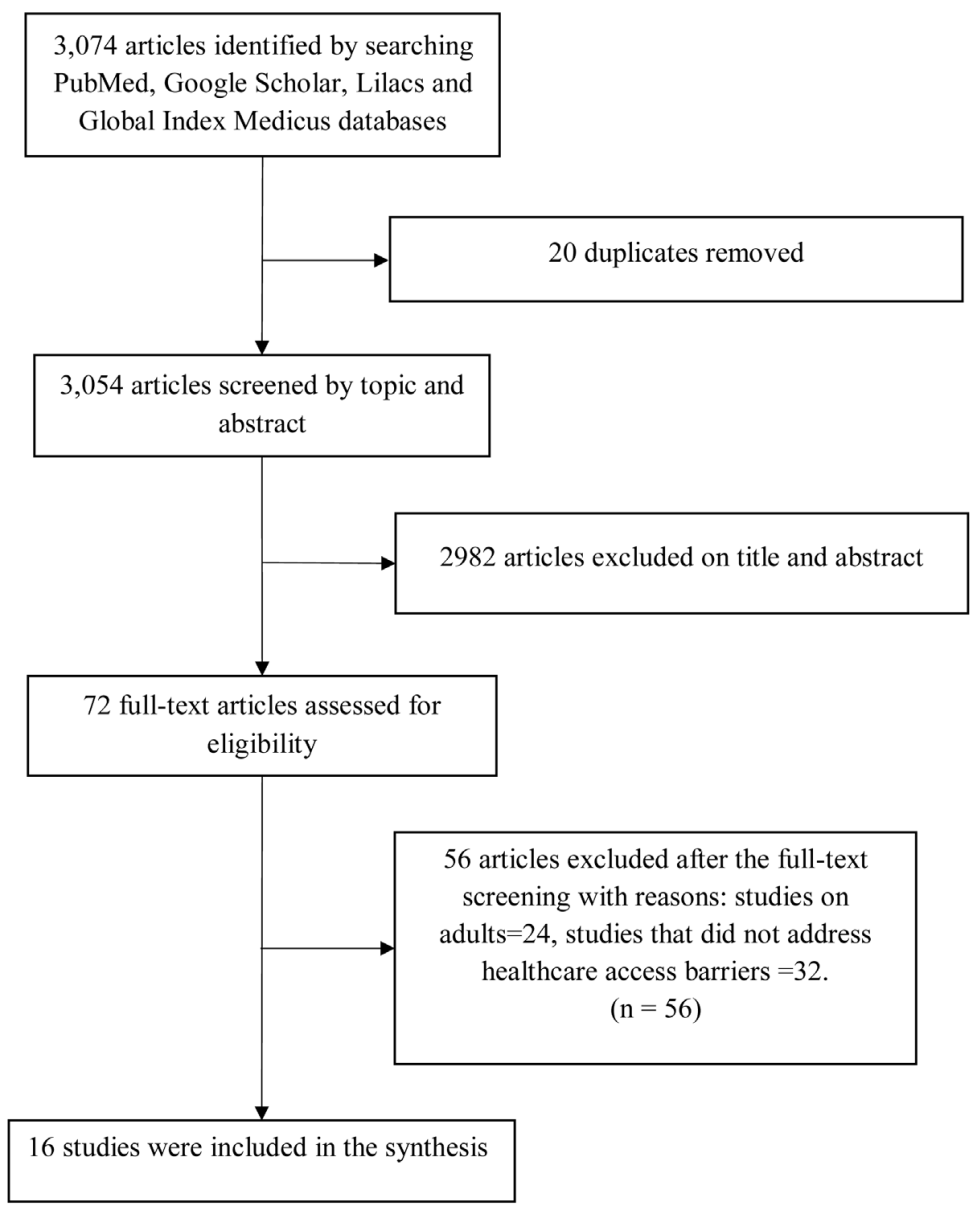
PRISMA flow diagram of the study selection process.

The database searches yielded 3,074 reports, of which 16 were eligible for the final analysis ( Figure 3). Most (87.5%) studies were conducted in Africa while the rest originated from Asia. Over 60% of these studies originated from LMICs while the remainder (<40%) came from low-income countries (LIs) and upper-middle-income countries (UMICs), respectively. There were more community-based studies (56.3%) compared with hospital-based reports (43.7%), and more cross-sectional studies (43.7%) than cohort studies (12.5%) ( Table 2). There were six (37.5%) studies on epilepsy, three (18.8%) studies on hearing impairment, two (12.2%) studies on cognitive impairment/intellectual disability, one (6.3%) study on vision impairment, and four (25.0%) studies in more than one domain of NI ( Table 3).

**Table 2.** Classification of the studies included by economy level and study setting.

Classification		Number of Studies (n=16)	The total sample size of participants in the study (n=7474)
Continent	Asia	2 (12.50%)	5,017
	Africa	14 (87.50%)	2,457
World Bank country income classification	Low-income	3 (18.75%)	631
	Low-middle-income	10 (62.5%)	1,483
	Upper-middle-income	3 (18.75%)	5,360
Study design	Cohort	2 (12.50%)	5,137
	Cross-sectional	7 (43.75%)	1,794
	Qualitative	7 (43.75%)	543
Setting	Community	8 (56.25%)	6,038
	Hospital	7 (43.75%)	1,436

**Table 3.** General characteristics of studies eligible for inclusion in the systematic review.

First Author & year of Publication	Country	Study design	Domain studied	Type of services discussed
Alloh *et al.*, 2009	Cote d’Ivore	Cross-sectional	Disabilities	Rehabilitative
Alrasheed *et al.*, 2018	Sudan	Qualitative	Vision	Preventive
Bassili *et al.*, 2002	Egypt	Cross-sectional	Epilepsy	Treatment/management
Burke *et al.*, 2017	Senegal	Qualitative	Disabilities	Preventive
Bright *et al.*, 2017	Malawi	Cohort	Hearing	Treatment/management
Carter *et al.*, 2012	Kenya	Qualitative	Epilepsy	Treatment/management
Gobrial, 2012	Egypt	Cross-sectional	Intellectual	Rehabilitative
He *et al.*, 2017	China	Retrospective cohort	Intellectual	Rehabilitative
Kirabira *et al.*, 2018	Uganda	Cross-sectional	Epilepsy	Treatment/management
Khoza-Shangase, 2019	South Africa	Qualitative	Hearing	Rehabilitative
Mbuba *et al.*, 2012	Kenya	Qualitative	Epilepsy	Treatment/management
Merugumala *et al.*, 2017	India	Qualitative	Hearing	Rehabilitative
El Sharkawy *et al.*, 2006	Kenya	Qualitative	Epilepsy	Treatment/management
Tataryn *et al.*, 2017	Malawi	Cross-sectional	Disabilities	Preventive
Wagner *et al.*, 2016	South Africa	Cross-sectional	Epilepsy	Treatment/management
Yousafzai *et al.*, 2005	Uganda, Rwanda	Qualitative	Disabilities	Preventive

### Healthcare for children with neurological impairment

Treatment/management services were sought in seven (43.8%) studies, rehabilitative care in five (31.3%) studies, and preventive care in four (25.0%) studies ( Table 3). Antiepileptic drugs used in the management of seizures included phenobarbital ( Bassili *et al.*, 2002; Mbuba *et al.*, 2012), phenytoin ( Bassili *et al.*, 2002), sodium valproate ( Bassili *et al.*, 2002), and carbamazepine ( Bassili *et al.*, 2002; Mbuba *et al.*, 2012). Bassili and colleagues also identified the use of electroencephalogram (EEG) and computed tomography for diagnosis or classification of epilepsy ( Bassili *et al.*, 2002). None of the epilepsy reports identified the use of surgical services. Preventive services reported in three studies included the screening of HIV/AIDS ( Yousafzai *et al.*, 2005), the provision of contraceptives for adolescents with disabilities ( Burke *et al.*, 2017), and screening services for children with hearing impairment ( Bright *et al.*, 2017). Rehabilitation for hearing impairment included the provision of assistive hearing devices ( Merugumala *et al.*, 2017) while children with intellectual disabilities sought strength training, ambulation and speech therapy care from occupational therapy, physical therapy and mental health departments ( Gobrial, 2012; He *et al.*, 2017).

### Barriers for healthcare (quantitative analysis)

**Figure 4.**
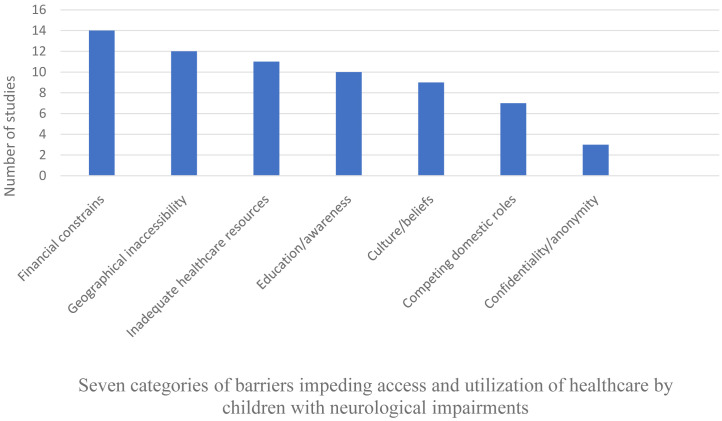
Number of studies reporting each category of barriers as classified in the analysis of the systematic review.

The main factors limiting accessing/utilization of healthcare services in >50% of the studies were financial constraints (N=14, 87.5%), geographical inaccessibility (N=12, 75%), inadequacy of healthcare resources (N=11, 68.8%), inadequate education/awareness (N=10, 62.5%), and prohibitive culture/beliefs (N=9, 56.3%). Factors reported in less than half of the studies were competing domestic roles (N=3, 18.6%), and a lack of confidentiality/anonymity (N=2, 12.5%), as illustrated in Figure 4. We identified very few reports originating from outside Africa which prevented statistical comparisons of individual reports by continents and economic level ( Table 4).

**Table 4.** Barriers limiting healthcare access classified by continents and levels of income.

Barriers	Continent		Economic level		
	Africa	Asia	LMI	LI	UMIC
Financial constrains	12 (85.7%)	2 (14.3%)	8 (57.1%)	3 (21.4%)	3 (21.4%)
Geographical accessibility	11 (91.7%)	1 (8.3%)	8 (66.6%)	3 (25%)	1 (8.3%)
Inadequate healthcare resources	10 (90.9%)	1 (9.1%)	8 (72.7%)	1 (9.1%)	2 (18.1%)
Inadequate education/awareness	8 (80%)	2 (20%)	7 (70%)	2 (20%)	1 (10%)
Culture/beliefs	7 (87.5%)	1 (12.5%)	5 (62.5%)	2 (25%)	1 (12.5%)
Competing domestic roles	2 (66.6%)	1 (33.3%)	2 (100%)	-	-
Confidentiality/anonymity	2 (100%)	-	2 (100%)	-	-

Abbreviations: LMI (low-middle-income), LI (low-income), UMIC (upper-middle-income).

### Barriers for healthcare (qualitative analyses)

***Financial constraints.*** Most caregivers could not afford the recurrent costs for antiepileptic drugs (AEDs) ( Carter *et al.*, 2012; El Sharkawy *et al.*, 2006; Kirabira *et al.*, 2018). "This hospital is good but sometimes you go there, get examined and prescribed for drugs and you need money for those drugs. So if you don't have money, then you just remain with the illness", reported a caregiver for a patient with epilepsy from a rural village in Kenya ( Carter *et al.*, 2012). Secondly, transportation costs hindered poorer families from accessing healthcare ( Carter *et al.*, 2012; Merugumala *et al.*, 2017; Tataryn *et al.*, 2017). A caregiver from India explained, “I know the center didn't ask for money, but it is the effort to get here right? We have to pay for bus fare and that's a lot for me. So, I left it even when they called us back” ( Merugumala *et al.*, 2017). Indirect costs of healthcare included loss of work-time ( Wagner *et al.*, 2016), loss of income ( Bright *et al.*, 2017), and diversion of limited family resources for treatment ( Carter *et al.*, 2012).

***Geographical access.*** The main geographical factors limiting access were unfavourable terrain ( Bright *et al.*, 2017), and distance decay ( Burke *et al.*, 2017; Carter *et al.*, 2012; El Sharkawy *et al.*, 2006; Mbuba *et al.*, 2012; Tataryn *et al.*, 2017). Unsuitable infrastructural design such as the absence of ramps, unfavourable public transport systems, and a lack of wheelchairs were also identified ( Merugumala *et al.*, 2017). “The journey itself is difficult because my son cannot walk yet, so if I don't find an auto-rickshaw, I have to carry him all the way down the long road to the center. That is very tiring especially in the summer heat, but what else can I do?”, complained a caregiver from India ( Merugumala *et al.*, 2017).

***Availability of healthcare resources.*** About 70% of the reports cited either an inadequate number of healthcare facilities, understaffing, or a lack of equipment and medication. Patients had difficulties in getting an appointment in public hospitals as there were few trained experts in neurology ( Alloh *et al.*, 2009; Alrasheed *et al.*, 2018). Poor diagnostic equipment and the unavailability of AEDs in healthcare facilities were common for epilepsy patients ( Carter *et al.*, 2012). Screening programs for children with hearing impairment were unavailable and rehabilitation facilities for intellectual disability were few and were located in urban centres ( Gobrial, 2012; Khoza-Shangase, 2019; Merugumala *et al.*, 2017).

***Education/awareness.*** Some caregivers lacked information on the causes and treatment of epilepsy ( Carter *et al.*, 2012), and about the existence of healthcare services ( Alrasheed *et al.*, 2018; Burke *et al.*, 2017; El Sharkawy *et al.*, 2006; Merugumala *et al.*, 2017; Tataryn *et al.*, 2017). Lack of health education programs was reported by caregivers for patients with epilepsy and visual impairments, respectively ( Alrasheed *et al.*, 2018; Bassili *et al.*, 2002). Health illiteracy limited caregivers’ awareness about the availability of specialist services and delayed or hindered the diagnosis of hearing impairment and management of children with intellectual disability ( He *et al.*, 2017; Merugumala *et al.*, 2017). Parents and health workers could not communicate with deaf adolescents using sign language and print material in health campaigns was not adapted for blind individuals ( Yousafzai *et al.*, 2005).

***Culture/beliefs.*** There were misconceptions about the cause of epilepsy where animistic beliefs were strongly held. “It is said that it is witchcraft. She was bewitched, that is according to our customs. That is when you will go to a *mganga* [traditional health practitioner] because you want to untrap them”, explained a grandmother to a child with epilepsy in Kenya ( Carter *et al.*, 2012). Alternative care including consultation of traditional health practitioners (THP) was preferred to biomedical care for childhood eye diseases ( Alrasheed *et al.*, 2018). Societal stigma hindered access to AEDs by children with epilepsy ( Kirabira *et al.*, 2018), as well as access to contraception by adolescents with disabilities ( Yousafzai *et al.*, 2005), and HIV/AIDS testing among adolescents with physical impairments ( Burke *et al.*, 2017). The dominant role of family elders in health-related decisions played a pivotal role where the grandmothers believed that deafness would resolve spontaneously ( Merugumala *et al.*, 2017). 

***Competing domestic roles, and lack of confidentiality.*** Three papers reported that childcare and other competing roles such as home care for a sick relative were given a higher priority over the healthcare for a child with a disability ( Bright *et al.*, 2017; Merugumala *et al.*, 2017; Tataryn *et al.*, 2017). Two studies ( Burke *et al.*, 2017; Yousafzai *et al.*, 2005) identified the lack of confidentiality and privacy of personal information as patients with disabilities needed to be accompanied by caregivers during consultations.

## Discussion

Most studies assessing access to healthcare for children with NI focused on epilepsy, with fewer reports on the other forms of NI. The main form of care reported for epilepsy patients was the management of seizures using AEDs; there was a lack of newer AEDs and other treatment options such as surgery. Preventive and rehabilitative services were less common in the reports from Africa. Overall, the main factors hindering access to healthcare by children with NI were financial constraints, geographical inaccessibility, inadequate healthcare resources, a lack of education or awareness, and prohibitive culture, respectively. Other important but less frequently reported factors were competing for domestic roles for caregivers, and a lack of confidentiality. Most studies originated from Africa and fewer studies were identified from other LMICs setting complicating valid comparison by continent and level of economic development.

Children with NI from impoverished families could not afford out-of-pocket payments for healthcare, a problem compounded by a lack of health insurance. Many families were forced to neglect healthcare to meet more pressing basic needs such as food and shelter. These can be addressed by expanding the scope of health insurance coverage to reduce out-of-pocket payments for healthcare in LMICs. Children with epilepsy could not regularly access AEDs, a common challenge in developing countries that can be solved by establishing community-based services, outreach programs and reducing the prices of AEDs in settings where they are costly. Indirect costs such as loss of work time have a significant economic impact on these families as time intended to be spent earning an income is used to care for their children. A cycle of poverty and disability might explain the inability of most caregivers to afford expensive primary care for their children ( Banks *et al.*, 2017). Wilmshurst and colleagues ( Wilmshurst *et al.*, 2014), affirm the finding that epilepsy management is expensive, and the cost of healthcare is unaffordable for many impoverished families. Additional expenses such as public transport and indirect costs such as loss of income were observed in previous studies ( Eide *et al.*, 2015).

Inadequate healthcare resources were common in most African studies. The lack of specialist services for visually impaired children reflects a previously reported shortage of 3.7 ophthalmologists per one million people in LMICs, a figure which is substantially low compared to 76.2 per million people in high-income countries ( Resnikoff *et al.*, 2020). This shortage can be resolved by investing in training and employing competent ophthalmologists. Lack of information and inadequate communication is a great challenge in LMICs. Ineffective communication between healthcare providers and patient/caregiver has previously been studied ( Maloni *et al.*, 2010), and patients and caregivers with NI would benefit from effective and clear communication from service providers, including the use of sign language for the deaf. Proper information packaging, effective doctor-patient communication, and further investment in health promotion campaigns might create and sustain awareness about neurodisability and healthcare ( WHO, 2018). It is also well-established that maternal education is strongly associated with the use of health services ( Armar-Klemesu *et al.*, 2000), while illiteracy of caregivers presents difficulties in understanding instructions from care providers ( Crabtree, 2007). Also, health education might play a critical role in supporting the previously suggested community-based outreach programs.

As seen in a previous study ( Eide *et al.*, 2015) poor terrain and long geographical distance significantly reduced the likelihood that children with NI from remote areas were able to access healthcare. Also, a lack of environmental modification to cater to those with physical impairment limited access to healthcare in some studies. For instance, there was a shortage of wheelchairs, and public transport systems and hospital facilities lacked provisions for people with physical disabilities. Distance decay, a phenomenon where service utilization reduces with increasing geographical distances from the healthcare facilities can be addressed through decentralizing healthcare and equipping rural health facilities with appropriate healthcare resources. Additionally, community-based rehabilitation has been recommended to complement the care provided by the existing healthcare systems ( Iemmi *et al.*, 2015). While the convention for the rights of people with disabilities, based on the International Classification of Functioning, Disability and Health (ICF), advocates for modification of the environment for the welfare of those with disabilities ( WHO, 2001; WHO, 2011), these recommendations have not been implemented in most LMICs. Governments must ensure disability mainstreaming in existing and future infrastructural development (such as the construction of ramps) and partnership with non-governmental/private entities in providing other forms of support (e.g. wheelchairs) for children with disabilities.

In terms of culture and beliefs, our results are similar to those from a Turkish study ( Diken, 2006), where mothers who perceived child disability to be a result of curses were more likely to seek traditional interventions. Misconceptions and animistic beliefs on the cause of NI were strongly associated with visiting THP. There is a need to integrate THP into formal healthcare alongside strengthening community-based rehabilitation ( Krah *et al.*, 2018). Attitudes from the patient/caregiver, healthcare provider, or societal perspectives were associated with decisions made by primary caregivers or children with NI regarding healthcare. For example, a lack of altruism and discrimination by healthcare providers, and a lack of privacy was observed on sexual and reproductive care for adolescents with disability ( Banks *et al.*, 2017; Eide *et al.*, 2015). Education and training of healthcare providers on equality and diversity are imperative to address the discrimination in the context of physician-patient-caregiver relationships. There is also a need to train non-existent specialities ( Bunning *et al.*, 2014) and capacity building of existing healthcare staff to be sustained ( Maloni *et al.*, 2010).

### Strengths and limitations

There were few studies identified by our searches, with most reports from Africa and none from South America. This will affect the generalizability of our findings, and specific studies are needed from unrepresented settings. Also, there were few studies on care for children with cognitive, hearing, motor, and vision impairments, possibly because few such studies exist for these impairments in paediatric populations in LMICs. Both quantitative and qualitative studies were eligible for analysis, but study design variability prevented the utility of purely qualitative or quantitative methods in this review. While children and adolescents represent a broad range of age-groups possibly with different factors influencing the utility of healthcare, there was no standard reporting of barriers in the primary studies reviewed, which should be standardized in future working groups by expert panels or task forces. However, to the best of our knowledge, this is the first systematic review to synthesize evidence of barriers limiting access and utilization of preventive, curative, and rehabilitative care by children and adolescents with NI in multiple domains in LMICs.

## Conclusion

Financial constraints, geographic inaccessibility, inadequate healthcare resources, poor communication/awareness, and cultural barriers were the most ubiquitous barriers limiting access and utilization of healthcare services by children with NI. There were more studies on epilepsy, and the use of preventive and rehabilitative care was less common. Expanding health insurance coverage, improving infrastructure with the decentralization of healthcare, and adequate training and staffing of care facilities, combined with investment in structured health promotion are fundamental steps towards addressing these challenges.

## Data availability

The data referenced by this article are under copyright with the following copyright statement: Copyright: ï¿½ 2021 Mwangi LW et al.

Data associated with the article are available under the terms of the Creative Commons Attribution Licence, which permits unrestricted use, distribution, and reproduction in any medium, provided the original data is properly cited. 

All data underlying the results are available as part of the article and no additional source data are required.

### Reporting guidelines

Harvard Dataverse: PRISMA checklist for ‘Barriers to access and utilization of healthcare by children with neurological impairments and disability in low-and middle-income countries: A systematic review’. https://doi.org/10.7910/DVN/H2V167 ( Abuga *et al.*, 2021).

Data are available under the terms of the Creative Commons Attribution 4.0 International license (CC-BY 4.0).
