# Supplementary material for: Barriers to access and utilization of healthcare by children with neurological impairments and disability in low-and middle-income countries: a systematic review
Source: Wellcome Open Res. 2022 Jan 24;6:61. Originally published 2021 Mar 18. [Version 2] doi: 10.12688/wellcomeopenres.16593.2 (PMC8902259; doi:10.12688/wellcomeopenres.16593.2)
Supplement: Peer review history

# Peer review: Reviewer response for version 2

**DOI:** 10.21956/wellcomeopenres.19467.r48191

**Published:** 2022-03-07

**Authors:** Samson Gwer

**Affiliations:** 1 School of Medicine, Kenyatta University, Nairobi, Kenya; 2 Afya Research Africa, Nairobi, Kenya

This is a greatly improved draft.

 The authors provide quality scores for the included studies. It would be great if they can complement this with a tabulation of the critical appraisal results, provided as supplementary material if there is a publication limit to the number of tables.

 The authors should consider a statement on the role of the co-authors in undertaking the systematic review.

Are the rationale for, and objectives of, the Systematic Review clearly stated?

Partly

Is the statistical analysis and its interpretation appropriate?

Partly

Are sufficient details of the methods and analysis provided to allow replication by others?

Partly

Are the conclusions drawn adequately supported by the results presented in the review?

Partly

Reviewer Expertise:

Child Neurology; Health Systems

I confirm that I have read this submission and believe that I have an appropriate level of expertise to confirm that it is of an acceptable scientific standard.

# Peer review: Reviewer response for version 2

**DOI:** 10.21956/wellcomeopenres.19467.r48192

**Published:** 2022-02-02

**Authors:** Helen McConachie

**Affiliations:** 1 Population Health Sciences Institute, Newcastle University, Newcastle, UK

This important systematic review has been improved by the authors, and they have responded appropriately to the comments I made on the original version. I still would have liked to know what use was made of the quality rating (e.g. whether less weight was given to findings from papers judged to be of low quality). However, it is not necessary for this to be addressed in a further revision. This paper will, I hope, be helpful to service commissioners and providers in considering how to overcome barriers for families in accessing and utilising services for children with neurological impairments and disability.

Are the rationale for, and objectives of, the Systematic Review clearly stated?

Partly

Is the statistical analysis and its interpretation appropriate?

Not applicable

Are sufficient details of the methods and analysis provided to allow replication by others?

Partly

Are the conclusions drawn adequately supported by the results presented in the review?

Yes

Reviewer Expertise:

NA

I confirm that I have read this submission and believe that I have an appropriate level of expertise to confirm that it is of an acceptable scientific standard.

# Peer review: Reviewer response for version 1

**DOI:** 10.21956/wellcomeopenres.18287.r44564

**Published:** 2021-07-05

**Authors:** Samson Gwer

**Affiliations:** 1 School of Medicine, Kenyatta University, Nairobi, Kenya; 2 Afya Research Africa, Nairobi, Kenya

In reviewing this paper, I take note of comments and suggestions by the first reviewer raising valid issues which I agree need to be addressed by the authors.

 The systematic review analyzes and summarizes different types of studies with different levels of evidence. There needs to be a clearer articulation of the mixed methods review process that has likely been used in conducting the review. This should include an explicit description of the data extraction and data synthesis processes used for the qualitative and quantitative types of studies

 The authors indicate that they assessed the quality of the included studies but they have not provided a summary of the assessment. There is scanty information about the included studies - this is important for transparency. The reader should be able to judge that the results presented are indeed the product of an unbiased analysis of considered and included studies.

 I also take note of a minor item in the abstract for clarification: What do the authors mean by “Survivors of Childhood Mortality” in the first statement on the abstract?

Are the rationale for, and objectives of, the Systematic Review clearly stated?

Partly

Is the statistical analysis and its interpretation appropriate?

Partly

Are sufficient details of the methods and analysis provided to allow replication by others?

Partly

Are the conclusions drawn adequately supported by the results presented in the review?

Partly

Reviewer Expertise:

Child Neurology; Health Systems

I confirm that I have read this submission and believe that I have an appropriate level of expertise to confirm that it is of an acceptable scientific standard, however I have significant reservations, as outlined above.

# Response

**Published:** 2022-01-10

**Authors:** Jonathan Abuga

**Affiliations:** Kemri-Wellcome Trust Research Programme, Kilifi, Kenya

Thank you very much for reviewing this paper and inviting us to address your comments and suggestions. We have addressed the comments and made a point-by-point response to each one of them as below.

**Comment #1**

 In reviewing this paper, I take note of comments and suggestions by the first reviewer raising valid issues which I agree need to be addressed by the authors.

***Reply:*** *Thank you for noting the valid comments and suggestions given by the first reviewer. We have addressed your comments and suggestions, including those given by the first reviewer, and revised the manuscript appropriately.*

**Comment #2**

 The systematic review analyses and summarizes different types of studies with different levels of evidence. There needs to be a clearer articulation of the mixed methods review process that has likely been used in conducting the review. This should include an explicit description of the data extraction and data synthesis processes used for the qualitative and quantitative types of studies.

***Reply*** *: We have provided additional information for quantitative and qualitative synthesis approaches on pages 7-8 of the revised paper. We’ve also described the list of variables that were extracted and the methods which were used in the data synthesis for qualitative and quantitative studies included in this review.*

**Comment #3**

 The authors indicate that they assessed the quality of the included studies, but they have not provided a summary of the assessment.

***Reply:*** *Thank you for this important observation, which was also noted by the first reviewer. We now provide a summary of the assessment of the quality of studies in the first paragraph of the Results section, on page 8.*

**Comment #4**

 There is scanty information about the included studies - this is important for transparency. The reader should be able to judge that the results presented are indeed the product of an unbiased analysis of considered and included studies.

***Reply:*** *We have now provided additional information about the included studies (Tables 1-4) to allow the readers to make an unbiased assessment of the results.*

**Comment #5**

 I also take note of a minor item in the abstract for clarification: What do the authors mean by “Survivors of Childhood Mortality” in the first statement on the abstract?

***Reply:*** *We intended to refer to children who survive the first five years of life in LMICs. For clarity, we have revised the sentence which now reads as follows. “Neurological impairments (NI) and disability are common among children residing in low-and middle-income countries (LMICs).*

# Peer review: Reviewer response for version 1

**DOI:** 10.21956/wellcomeopenres.18287.r43320

**Published:** 2021-04-06

**Authors:** Helen McConachie

**Affiliations:** 1 Population Health Sciences Institute, Newcastle University, Newcastle, UK

This systematic review addresses an important topic, aiming to identify barriers which affect access to healthcare for children with neurodisability and their families. Identification of barriers allows the proposal and development of strategies to overcome such barriers, and the authors include this in the Discussion.  The review identifies seven categories of barriers from the literature selected; it is likely to be the first systematic review of this topic. Thus the findings are useful for the field.

 However, there are a number of limitations to the methods of the review which limit its comprehensiveness. This does not necessarily invalidate the findings concerning types of barriers, though others of relevance may have been missed. However, it does create bias in some of the authors' statements concerning how frequently different types of conditions or disorders would be represented in the literature, and such references should be rewritten (e.g. the first sentence of the Discussion, and the second sentence of the Conclusion). Of limitations, first, the authors considered only papers published in English, in only four databases, and did not include grey literature or searches by key author surnames. Second, the search terms for neurodisability were limited mostly to 'impairment', i.e. apart from epilepsy. In the span of time searched (1990-2019), before the publication of the ICF in 2001, a range of etiological and disability terms would have been used. For example, a search on 'cerebral palsy' (not just 'motor impairment') would have yielded a number of additional papers examining barriers to service use in LMICs. It is not clear that the authors did a preliminary check that key expected papers were found by their search strategy, and the resulting focus on epilepsy studies in their results is not surprising. Third, two reviewers independently screened papers on title and abstract for eligibility, but there is no statement as to whether there was any check on agreement at this crucial stage. Fourth, the authors state they examined the quality of studies but how this information was used is not reported. Fifth, there is inadequate description of how the seven themes to classify barriers were derived. Was this drafted in advance, or from the data extracted? Was there any check on the validity/reliability of the classification? What type of evidence was used - were single quotes from a paper sufficient, or was the paper itself required to have presented categories?  Thus not enough information is presented so that the systematic review could be repeated by others. Obviously, the authors cannot redo the review at this stage, but a fuller acknowledgement of limitations in the Discussion is necessary.

 Overall, the review takes a rather narrow focus, and some of the writing seems to reflect a medical mind-set rather than considering the wider needs of the child in the family and community. For example, the first sentence of the Abstract describes a focus on "survivors of childhood mortality" rather than reflecting the multifactorial background of child neurodisability. Likewise, the first paragraph of the Introduction emphasises hospitalisation from accidents or infection and only moves to prevention and rehabilitation in the second paragraph. The Introduction does not present a very thorough justification of the need for the review, simply saying the evidence concerning barriers is fragmented. To my mind a broader introduction would have been beneficial - for example, I was surprised not to see any reference to the influential overview article by Patel *et al.* (2013 Arch Dis Child) ^1^ which also discussed barriers such as lack of evidence-based treatments and lack of identification of disorders.

 Figure 4 is unnecessary.

Are the rationale for, and objectives of, the Systematic Review clearly stated?

Partly

Is the statistical analysis and its interpretation appropriate?

Not applicable

Are sufficient details of the methods and analysis provided to allow replication by others?

Partly

Are the conclusions drawn adequately supported by the results presented in the review?

Yes

Reviewer Expertise:

Child disability; Systematic reviews

I confirm that I have read this submission and believe that I have an appropriate level of expertise to confirm that it is of an acceptable scientific standard, however I have significant reservations, as outlined above.

## References

[1] : Improving access to care for children with mental disorders: a global perspective. *Arch Dis Child* .2013;98(5) : 10.1136/archdischild-2012-302079 323-7 10.1136/archdischild-2012-302079 23476001PMC3672840

# Response

**Published:** 2022-01-10

**Authors:** Jonathan Abuga

**Affiliations:** Kemri-Wellcome Trust Research Programme, Kilifi, Kenya

Thank you very much for reviewing this paper and inviting us to address your comments and suggestions. We have addressed the comments and made a point-by-point response to each one of them as below.

**Comment #1:**

 This systematic review addresses an important topic, aiming to identify barriers that affect access to healthcare for children with neurodisability and their families. Identification of barriers allows the proposal and development of strategies to overcome such barriers, and the authors include this in the Discussion.  The review identifies seven categories of barriers from the literature selected; it is likely to be the first systematic review of this topic. Thus, the findings are useful for the field. However, there are a number of limitations to the methods of the review which limit its comprehensiveness. This does not necessarily invalidate the findings concerning types of barriers, though others of relevance may have been missed. However, it does create bias in some of the authors' statements concerning how frequently different types of conditions or disorders would be represented in the literature, and such references should be rewritten (e.g. the first sentence of the Discussion, and the second sentence of the Conclusion).

***Reply:*** *Thank you very much for appraising our work and for identifying potential limitations in the methods section for our attention. In our reply to your comments numbered 6-8 below, we respond to each specific question about the identification and classification of barriers and the reasons why we focussed on the selected categories*. *We hope that these revisions have improved the paper.*

**Comment #2:**

 The authors considered only papers published in English, in only four databases, and did not include grey literature or searches by key author surnames.

***Reply:*** *Thank you for pointing out this limitation, which we now discuss in the discussion of the revised manuscript. This was a systematic review whose focus was mostly on peer-reviewed articles. This systematic review approach gives equal chances of article identification to both key and new authors. We had intended to include non-English articles but struggled to get translation assistance for various foreign languages, a consistent challenge in many of our previous reviews. Our searches, however, identified a critical number of reports published in sub-Saharan Africa (SSA), which we think might be representative of this region. We have acknowledged this limitation in the strengths and limitations section of the revised manuscript (page 17, lines 338-352).*

**Comment #3:**

 Second, the search terms for neurodisability were limited mostly to 'impairment, i.e. apart from epilepsy. In the span of time searched (1990-2019), before the publication of the ICF in 2001, a range of etiological and disability terms would have been used. For example, a search on 'cerebral palsy' (not just 'motor impairment') would have yielded a number of additional papers examining barriers to service use in LMICs. It is not clear that the authors did a preliminary check that key expected papers were found by their search strategy, and the resulting focus on epilepsy studies in their results is not surprising

***Reply:*** *We did a preliminary check of the expected studies and the search strategies and identified most of the key studies that we were aware of at the time of the initial search. While we acknowledge that our search strategy might have missed some studies given the limitations in the search terms and the changes in commonly used terminology over the review period, we believe that some studies of cerebral palsy would have described motor deficits/impairments in the text field of these reports, and would be identified since we did not restrict search terms by title, abstract or text. W* *e, however, have conducted additional searches in the select databases using the additional search terms “OR cerebral palsy” for the same period and identified four additional papers that meet the inclusion criteria. Details of these four articles are now reflected in the methods, results, and discussion of the revised manuscript.***

**Comment #4:**

 The reviewers independently screened papers on title and abstract for eligibility, but there is no statement as to whether there was any check on the agreement at this crucial stage.

***Reply:*** *Thank you for this observation. At the screening stage, two authors (LM and JA) compared the list of reports identified by the searches as to whether they met the predefined inclusion criteria; all disagreements were resolved by consensus before proceeding to the next stage. This information is now provided on page 7, lines 113-114 of the revised manuscript.*

**Comment #5:**

 The author’s state they examined the quality of studies but how this information was used is not reported.

***Reply:*** *We acknowledge this oversight in our report. We now report the median percentage scores, for instance, 90% (range 37.5-90.0) for quantitative studies, based on the* *Joanna Briggs Institute (JBI) critical appraisal tools *in the results of the revised manuscript (see the first paragraph of the Results).**

**Comment #6:**

 There is an inadequate description of how the seven themes to classify barriers were derived. Was this drafted in advance, or from the data extracted?

***Reply:** Thank you for this important question. The seven themes into which we classified the barriers were based on data extracted from the first 10 articles meeting the inclusion criteria and were constantly updated with new emerging themes from the subsequent review of the remainder articles. For this reason, the categories (themes) we identified might only exclude other relevant categories not captured by our searches. We used a three-stage process to identify and classify barriers from the primary articles. In stage I, three reviewers (LM, EC, and JA) each independently identified the emerging categories in the data retrieved from the primary articles. In stage II, the three reviewers held a joint meeting to present and discuss the identified categories, each at a time. Some categories were merged, and others were created to include reviewers’ suggestions logically and comprehensively. In stage III, the reviewers held the final joint consensus meeting on a separate date to review the created categories before writing the final report. We also identified verbatim excerpts from the eligible primary qualitative studies to represent the voices of the caregivers about their perceptions of the barriers to care for their disabled children.* *The three-stage process is now described on pages 7 and 8, in the Methods section, and pages 12-13 in the Results section of the revised manuscript.*

**Comment #7:**

 Was there any check on the validity/reliability of the classification?

***Reply:*** *As highlighted in our response to comment # 6 above, we addressed the inter-observer validity/reliability of our classification approach in a three-step process. We think that there is room for improving this thematic framework in future reviews.*

**Comment #8:**

 What type of evidence was used - were single quotes from a paper sufficient, or was the paper itself required to have presented categories?  Thus, not enough information is presented so that the systematic review could be repeated by other by others. Obviously, the authors cannot redo the review at this stage, but a fuller acknowledgement of limitations in the Discussion is necessary.

***Reply:*** *As this was a systematic review to provide published data on the subject, single or multiple quotes identified in the primary article were sufficient; the report itself did not have to present the categories as defined by the reviewers as long as the barriers could be inferred from what was already provided. We provide this information in the Methods section (pages 7-8) of the revised manuscript. We also acknowledge the limitations of this approach in the Discussion.*

**Comment #9:**

 Overall, the review takes a rather narrow focus, and some of the writing seems to reflect a medical mindset rather than considering the wider needs of the child in the family and community. For example, the first sentence of the Abstract describes a focus on "survivors of childhood mortality" rather than reflecting the multifactorial background of child neurodisability. Likewise, the first paragraph of the Introduction emphasises hospitalisation from accidents or infection and only moves to prevention and rehabilitation in the second paragraph.

***Reply:*** *Thank you for raising this important comment. We acknowledge the multidimensional perspectives of disability and how these have evolved. We have revised the abstract and the introduction sections, following your recommendations, to include the multifactorial background to child neurodisability based on the ICF framework, without deviating from the focus of our review, which was to identify barriers limiting access and utilization of biomedical services, including preventive, curative and rehabilitative care.*

**Comment #10:**

 The Introduction does not present a very thorough justification of the need for the review, simply saying the evidence concerning barriers is fragmented. To my mind a broader introduction would have been beneficial - for example, I was surprised not to see any reference to the influential overview article by Patel *et al.* (2013 Arch Dis Child) ^1^ which also discussed barriers such as lack of evidence-based treatments and lack of identification of disorders.

***Reply*** *: Thank you for this suggestion. We have broadened the introduction to justify the need for this systematic review. We have also included ideas from the landmark overview article by Patel et al. considering the context of this work. Please see the revisions on pages 4 and 5 of the revised manuscript. * 

**Comment #11**:

 Figure 4 is unnecessary

***Reply*** *: Thank you for this observation. We have removed figure 4 and all references to it in the revised manuscript*
